# Alteration of the hypothalamic-pituitary-gonadal axis in estrogen- and androgen-treated adult male leopard frog, *Rana pipiens*

**DOI:** 10.1186/1477-7827-3-2

**Published:** 2005-01-10

**Authors:** Pei-San Tsai, Ann E Kessler, Jeremy T Jones, Kathleen B Wahr

**Affiliations:** 1 Department of Integrative Physiology and the Center for Neuroscience, University of Colorado, Boulder, CO 80309-0354, USA

## Abstract

**Background:**

Gonadal steroids, in particular 5 alpha-dihydrotestosterone (DHT) and 17 beta-estradiol (E2), have been shown to feed back on the hypothalamic-pituitary-gonadal (HPG) axis of the ranid frog. However, questions still remain on how DHT and E2 impact two of the less-studied components of the ranid HPG axis, the hypothalamus and the gonad, and if the feedback effects are consistently negative. Thus, the goal of the study was to examine the effects of DHT and E2 upon the HPG axis of the gonadally-intact, sexually mature male leopard frogs, Rana pipiens.

**Methods:**

*R. pipiens *were implanted with silastic capsules containing either cholesterol (Ch, a control), DHT, or E2 for 10 or 30 days. At each time point, steroid-induced changes in hypothalamic GnRH and pituitary LH concentrations, circulating luteinizing hormone (LH), and testicular histology were examined.

**Results:**

Frogs implanted with DHT or E2 for 10 days did not show significant alterations in the HPG axis. In contrast, frogs implanted with hormones for 30 days had significantly lower circulating LH (for both DHT and E2), decreased pituitary LH concentration (for E2 only), and disrupted spermatogenesis (for both DHT and E2). The disruption of spermatogenesis was qualitatively similar between DHT and E2, although the effects of E2 were consistently more potent. In both DHT and E2-treated animals, a marked loss of all pre-meiotic germ cells was observed, although the loss of secondary spermatogonia appeared to be the primary cause of disrupted spermatogenesis. Unexpectedly, the presence of post-meiotic germ cells was either unaffected or enhanced by DHT or E2 treatment.

**Conclusions:**

Overall, these results showed that both DHT and E2 inhibited circulating LH and disrupted spermatogenesis progressively in a time-dependent manner, with the longer duration of treatment producing the more pronounced effects. Further, the feedback effects exerted by both steroid hormones upon the HPG axis were largely negative, although the possibility exists for a stimulatory effect upon the post-meiotic germ cells.

## Background

It is well established in mammals that gonadal steroid hormones are potent negative feedback regulators of the hypothalamic-pituitary-gonadal (HPG) axis. In ranid frogs, the first evidence supporting this notion came from a study on the bullfrog, *Rana catesbeiana*, in which gonadectomy elevated circulating gonadotropins, and estrogen and androgen replacement suppressed this elevation [1]. It was later shown that two gonadal steroids, 17β-estradiol (E_2_) and 5α-dihydrotestosterone (DHT), could directly target the pituitary to modulate the release of luteinizing hormone (LH) and follicle-stimulating hormone (FSH) in these frogs [2-4].

Despite the established role of DHT and E_2 _as feedback regulators of gonadotropin secretion in ranid frogs, there is still some confusion regarding the exact nature of these feedback effects. For example, in female and juvenile *R. catesbeiana*, DHT suppressed the post-gonadectomy rise in circulating gonadotropins [1], yet it enhanced the responsiveness of the pituitary to gonadotropin-releasing hormone (GnRH) [1,5], suggesting DHT is involved in both negative and positive feedback. On the other hand, in the leopard frog (*R. pipiens*), DHT had no effect on the post-castration rise in gonadotropin in males [2] but modestly stimulated pituitary responsiveness to GnRH, suggesting a role in only positive feedback. Although the effects of E_2 _were less variable, some conflicting data also exist. In both *R. catesbeiana *and *R. pipiens*, E_2 _consistently inhibited LH and FSH secretion both *in vivo *and *in vitro*, demonstrating a direct and powerful negative feedback effect of E_2 _at the level of the pituitary [1-3]. However, recent studies reported E_2 _treatment significantly stimulated the proliferation of primary spermatogonia (I SPG) in the green frogs, *R. esculenta *[6,7], suggesting an additional role of E_2 _in the positive feedback of the HPG axis.

Results from the previous studies revealed the complex nature in which estrogen and androgen feed back on the reproductive axis, and suggest that the nature of the feedback effects might vary depending on the species, sex, reproductive stage of the frogs used, and the duration of steroid hormones administered. Further, there are still significant gaps in our knowledge regarding how these two steroid hormones feed back on the other two components of the HPG axis, the hypothalamus and the gonad. Therefore, the goal of the present study is to understand how E_2 _and DHT impact the HPG axis in gonadally-intact male *R. pipiens*. We will do so by measuring parameters that reflect the function of the HPG axis, including hypothalamic GnRH, pituitary and circulating LH, and spermatogenic activity. Moreover, steroid treatments were administered over two periods, 10 days and 30 days, to examine if the nature of steroidal feedback effects remains consistent over time. These results should allow us to determine if DHT and E_2 _act consistently as negative feedback regulators at different levels of the HPG axis.

## Methods

### Animals

All experimental procedures were conducted in compliance with the animal protocol approved by the Institutional Animal Care and Use Committee at the University of Colorado. Mature male northern leopard frogs, *Rana pipiens*, were obtained from Carolina Biologicals (Burlington, NC) from April to July of 2004. Since the life histories of these animals were not entirely clear, all experiments were conducted within the three-month period to minimize the possible confounding effects of seasonality. As a control, we performed histological analysis on testes of representative frogs from every batch. When testicular histology was compared, little differences were observed among batches of frogs arriving at different times (data not shown). Frogs were kept under a 12L:12D photoperiod and fed live crickets every other day. All frogs were allowed to acclimate to the laboratory environment for at least one week before surgical implant.

### Surgical Implant

One-cm silastic capsules containing crystalline cholesterol (Ch: control), DHT, or E_2 _were prepared as previously described [8], with some minor modifications. Briefly, silastic tubing (outer diameter = 1.96 mm; inner diameter = 1.47 mm) was filled with 1 cm length of crystalline Ch, DHT, or E_2 _(Sigma, St Louis, MO) and sealed on both ends with silicon glue. Ch implant was widely used as a negative control for the experimental treatment of cholesterol-based compounds such as steroid hormones [8-11]. In a preliminary observation (data not shown), we noted no difference in the testicular morphology in animals that have lost Ch capsules compared to animals that have retained capsules throughout the 30-day period, indicating Ch had minimal effects on the reproduction of *R. pipiens*. Filled capsules were equilibrated in 0.6% saline for at least 48 hours before implant. For implant, frogs were anesthetized by immersion in 0.03% benzocaine. A small incision was made at the base of the left leg and a silastic capsule inserted subcutaneously. The incision was then closed with silk thread. Frogs were monitored for recovery from anesthesia and then returned to their respective holding tanks.

### Tissue Preparation

Ten or 30 days after implant, frogs were weighed and sacrificed by quick decapitation using a guillotine. Trunk blood was collected into heparinized tubes, centrifuged, and plasma stored at -70°C until the measurement of LH and steroid hormones by radioimmunoassays (RIAs). Testes were removed, their masses recorded, and immersion-fixed in Bouin's fixative overnight. Hypothalami were excised from the brain by four cuts: a coronal cut 1 mm rostral to the optic chiasm, a coronal cut on the caudal border of the optic tectum, and two sagittal cuts along the lateral margins of the median eminence. Hypothalami were flash-frozen on dry ice and stored at -70°C until extraction and the measurement of GnRH by RIA. Pituitary glands were removed, sonicated in 500 μl phosphate-buffered saline (PBS), and stored at -70°C until the measurement of LH by RIA. All carcasses were later inspected for the presence of the silastic capsule in the legs. Animals whose capsules were lost were excluded from data analysis.

### LH RIA

Plasma and pituitary LH levels were measured by a LH RIA developed for the bullfrog (*R. catesbeiana*) [12] and validated for *R. pipiens *[13]. The iodination stock, standard, and antiserum for the RIA were a generous gift of Dr. Paul Licht (University of California at Berkeley). The limit of detection was 0.1 ng/ml. The intra- and inter-assay coefficients of variation were 4.8% and 13.3%, respectively. Pituitary LH levels were normalized for protein content assessed by the Bradford protein assay (Bio-Rad Laboratories, Inc., Hercules, CA).

### Extraction of Hypothalami and GnRH RIA

Hypothalamic GnRH was extracted with 1 N HCl from the frozen tissues as previously described [11]. The recovery for hypothalamic extractions, assessed by the post-extraction counting of a known amount of [^125^I]GnRH added to representative homogenates prior to extraction, was 86%. GnRH RIA was carried out with an antiserum specific for the mammalian form of GnRH (R1245, provided by Dr. Terry Nett at the Colorado State University) using a protocol described in detail elsewhere [11,14,15]. The intra- and inter-assay coefficients of variation were 7.3% and 5.0%, respectively. Hypothalamic GnRH levels were normalized for protein content determined by the Bradford protein assay.

### Steroid Hormone RIAs

E_2 _and DHT RIAs were performed using the RIA kits from Diagnostic Systems Laboratories (Webster, TX). These RIA kits have been validated previously for the measurement E_2 _and DHT in *R. pipiens *[11]. The limits of detection were 6.5 pg/ml for the E_2 _RIA and 4 pg/ml for the DHT RIA. The intra- and inter-assay coefficients of variation were 5.3% and 4.9%, respectively, for the E_2 _RIA, and 3.1% and 8.4%, respectively, for the DHT RIA. Both RIAs are highly specific and cross-react minimally with other steroid hormones.

### Histology

After fixation, testes were dehydrated through ascending concentrations of ethanol, defatted in Histoclear, and embedded in paraffin. Thirteen-μm sections were cut on a rotary microtome, mounted on poly-L-lysine-coated slides, and stained with hematoxylin and eosin.

### Immunocytochemistry (ICC) of Proliferating Cell Nuclear Antigen (PCNA)

Testes were processed for ICC of PCNA, a cell cycle S phase marker, to identify I SPG and secondary spermatogonia (II SPG) undergoing cell proliferation [16]. Testicular sections, prepared as described above for histological staining, were deparaffinized in Histoclear, rehydrated through descending concentrations of ethanol, and immersed in Antigen Unmasking Solution (Vector Laboratories, Burlingame, CA) for 10 minutes at 90°C. After antigen retrieval, sections were washed with 1% hydrogen peroxide in 0.1 M PBS containing 0.4% Triton × 100 (PBST) for 10 minutes to quench the endogenous peroxidase activity, rinsed 5 times with PBST, and incubated for 48 hours at 4°C in PBST containing a monoclonal anti-PCNA antibody (Santa Cruz Biotechnology, Santa Cruz, CA; 1:500) and 4% normal sheep serum. After incubation, sections were washed with PBST and incubated with a biotinylated sheep-anti-mouse IgG (Jackson Laboratory, West Grove, PA; 1:400), washed, and incubated with the Vectastain ABC reagent (Vector Laboratories) for 1 hour. Sections were washed and the immunoreactivity visualized using diaminobenzidine as the chromagen. After the color reaction, sections were washed, counterstained with hematoxylin, dehydrated through ascending concentrations of ethanol, cleared in Histoclear, and coverslipped. Controls for ICC included the preadsorption of the primary antiserum with 20 μg/ml of recombinant human PCNA (Spring Bioscience, Fremont, CA) and the omission of the primary antiserum.

### Histological Analysis

Five to eight testes (each from a different animal) per treatment group were assessed for the following histological parameters: seminiferous tubule diameter, the number of I SPG per tubule, and the number of cysts within each tubule containing II SPG, primary spermatocytes (I SPC), or secondary spermatocytes (II SPC). These germ cells were defined according to Rastogi *et al*. [17]. To score the number of cysts containing II SPG, I SPC, and II SPC, sections stained with hematoxylin and eosin were used. To score the number of I SPG, which were scattered and more difficult to locate, sections processed for PCNA ICC were used. Specifically, PCNA-positive cells that were large, isolated, and located at the periphery of the cysts were scored as proliferating I SPG. This method allowed us to identify more I SPG than if morphological criteria were used alone. Since most spermatids and mature spermatozoa were not confined within the cysts and were therefore difficult to measure, these two germ cell types were not quantified. For each testis, five randomly selected tubules were sampled on a slide that had been coded to conceal the identity of the animal. Histological parameters from five tubules were averaged to give a mean for a single animal. Tubular diameters were measured using a calibrated ocular micrometer. All histological parameters were assessed by one individual blind to the identity of the slides.

### Statistical Analysis

Differences among groups were analyzed by the one-way analysis of variance (ANOVA) on log_10_-transformed data followed by the Tukey's post-hoc test. Differences were considered significant when *P *< 0.05.

## Results

Steroid hormone RIAs were performed to monitor circulating steroid hormone levels in Ch- and hormone-implanted animals. For animals implanted for 10 days, circulating E_2 _levels were 540 ± 110.5 (Ch group; n = 5) and 1966 ± 13.2 pg/ml (E_2 _group; n = 5), and circulating DHT levels were 0.9 ± 0.3 (Ch group; n = 4) and 37.5 ± 4 ng/ml (DHT group; n = 5). For animals implanted for 30 days, circulating E_2 _levels were 488 ± 370 (Ch group; n = 5) and 2255 ± 650 pg/ml (E_2 _group; n = 9), and circulating DHT levels were 2.1 ± 1.4 (Ch group; n = 4) and 24.6 ± 2.7 ng/ml (DHT group; n = 8).

**Figure 1:**
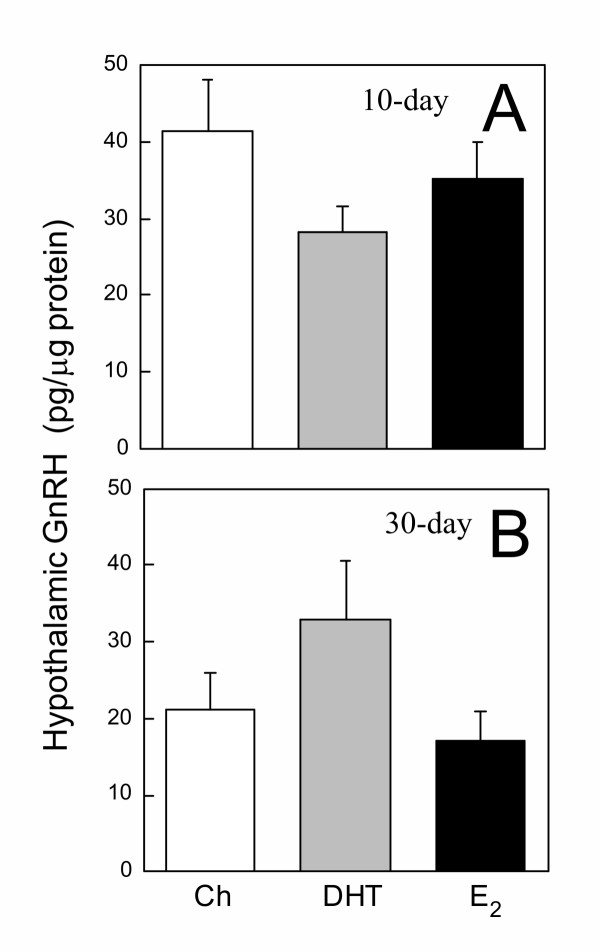
**Hypothalamic GnRH concentrations in implanted frogs. **Hypothalamic GnRH concentrations in frogs implanted for **(A) **10 days or **(B) **30 days with either Ch, DHT, or E_2_. No significant differences were observed among treatment groups in either 10- or 30-day-implanted animals. Each bar represents mean ± SEM. N = 6–11.+

**Figure 2:**
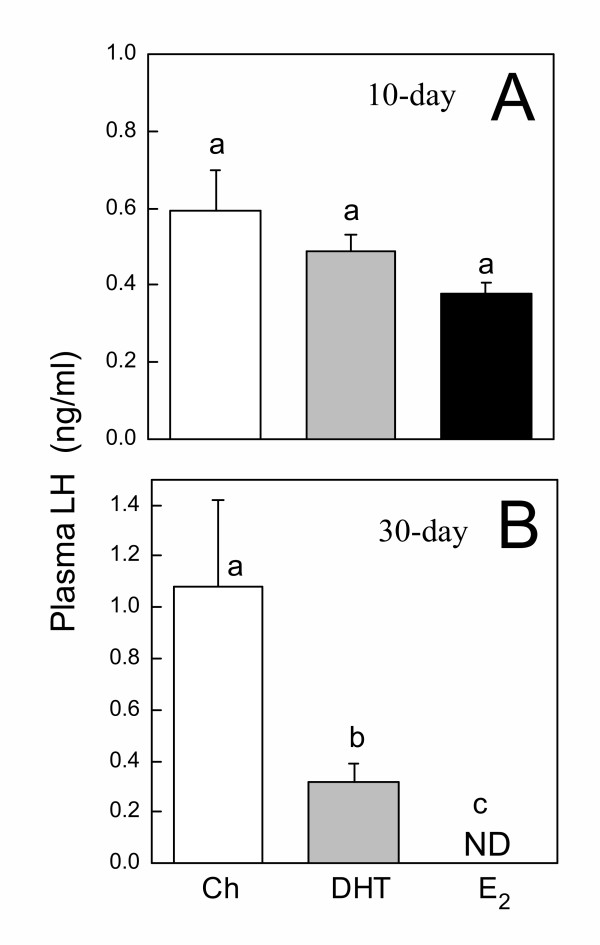
**Plasma LH levels in implanted frogs. **Plasma LH levels in frogs implanted for **(A) **10 days or **(B) **30 days with either Ch, DHT, or E_2_. Each bar represents mean ± SEM. Dissimilar letters indicate significant difference between groups. ND = not detectable. N = 7–10.

**Figure 3:**
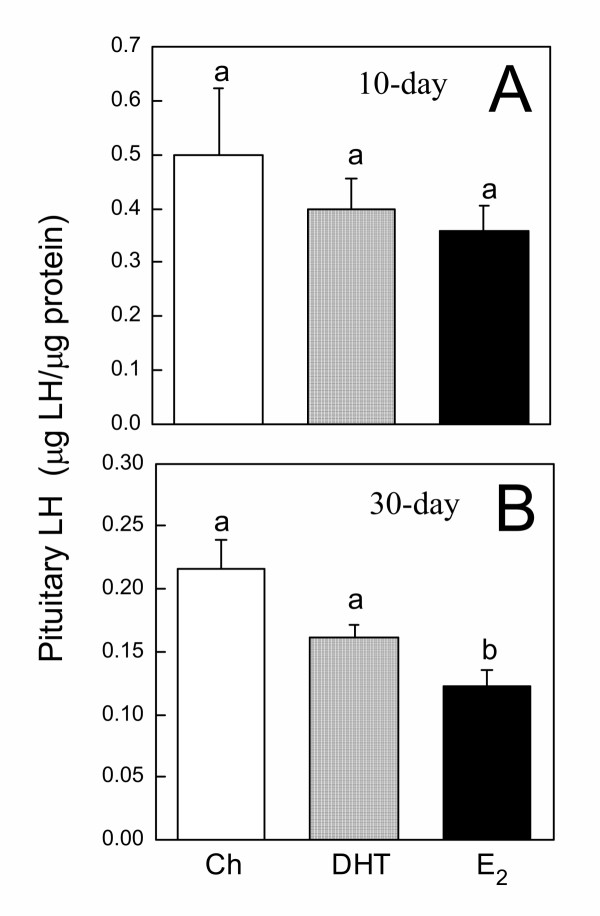
**Pituitary LH concentrations in implanted frogs. **Pituitary LH concentrations in frogs implanted for **(A) **10 days or **(B) **30 days with either Ch, DHT, or E_2_. Each bar represents mean ± SEM. Dissimilar letters indicate significant difference between groups. N = 7–10.

To assess the overall accumulation of GnRH in the hypothalami of control and steroid hormone-treated animals, hypothalami were removed, extracted, and measured for the concentration of GnRH. No significant differences in hypothalamic GnRH concentration were observed among Ch, DHT, and E_2 _groups implanted for 10 or 30 days (Fig. 1). In animals implanted for 10 days, plasma LH levels were not different among the treatment groups (Fig. 2A). However, in animals implanted for 30 days, both DHT and E_2 _significantly suppressed circulating LH (Fig. 2B). The suppressive effect of E_2 _was significantly more potent than DHT, with all E_2_-treated animals having undetectable levels of circulating LH (Fig. 2B). In 10-day-implanted frogs, no differences in pituitary LH concentration were observed among the treatment groups (Fig. 3A). In 30-day-implanted frogs, E_2_, but not DHT, significantly decreased the accumulation of LH in the pituitary (Fig. 3B).

**Figure 4:**
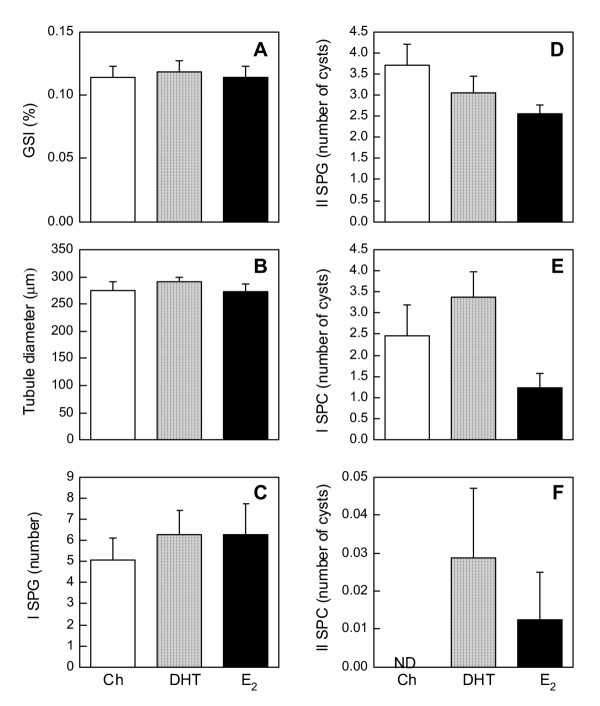
**Testicular function in frogs implanted for 10 days. **Measurements of testicular function in frogs implanted for 10 days with either Ch, DHT, or E_2_. **(A) **Average GSI, **(B) **average diameter of seminiferous tubules, **(C) **number of I SPG, and number of cysts containing **(D) **II SPG, **(E) **I SPC, and **(F) **II SPC were measured from 5–8 animals per treatment group. Each bar represents mean ± SEM. ND = not detectable. No significant differences were observed among treatment groups in any of the parameters measured.

**Figure 5:**
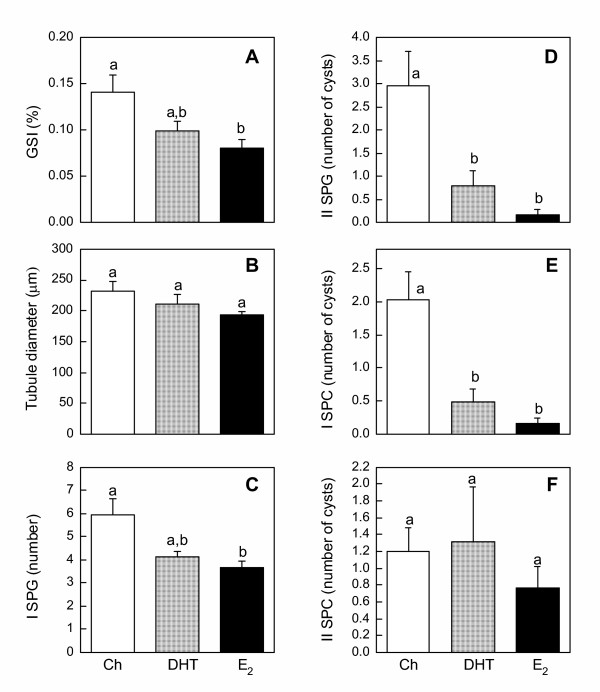
**Testicular function in frogs implanted for 30 days. **Measurements of testicular function in frogs implanted for 30 days with either Ch, DHT, or E_2_. **A) **Average GSI, **(B) **average diameter of seminiferous tubules, **(C) **number of I SPG, and number of cysts containing **(D) **II SPG, **(E) **I SPC, and **(F) **II SPC were measured from 5–6 animals per treatment group. Each bar represents mean ± SEM. Dissimilar letters indicate significant difference between groups.

**Figure 6:**
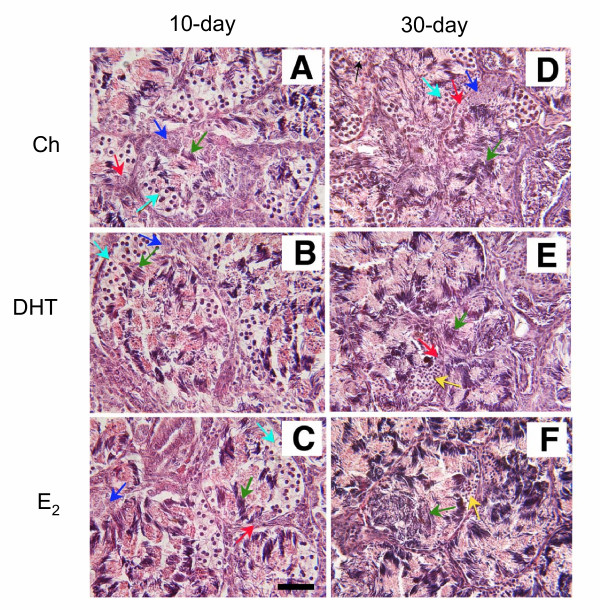
**Testicular histology of implanted frogs. **Representative testicular histology of frogs implanted with **(A, D) **Ch, **(B, E) **DHT, or **(C, F) **E_2_. **(A, B, C) **Testes from animals implanted for 10 days. **(D, E, F) **Testes from animals implanted for 30 days. Red arrow = I SPG; dark blue arrow = II SPG; light blue arrow = I SPC; yellow arrow = II SPC; green arrow = spermatozoa. Note DHT and E_2 _had no visible effects on testes of animals implanted for 10 days **(A, B, C)**. In contrast, DHT and E_2 _visibly altered the germ cell composition in animals implanted for 30 days **(D, E, F)**. Note the prominent spermatozoa and the absence of I SPC and II SPG in both DHT- and E_2_-treated testes **(E, F)**. Scale bar = 100 μm.

Testicular function was assessed by six parameters: the gonadosomatic index (GSI; [g testes mass/g body mass] × 100), the diameter of the seminiferous tubules, and the presence of four germ cell types (I SPG, II SPG, I SPC, II SPC) in the testes. Overall, no differences were observed in any of the six parameters among treatment groups in the 10-day-implanted animals (Fig. 4). However, in the 30-day-implanted animals, significant steroid-induced changes in the testes were seen. E_2 _significantly depleted the presence of I SPG, II SPG, I SPC, and reduced the GSI (Fig. 5), whereas DHT significantly reduced only the presence of II SPG and I SPC (Fig. 5). Neither steroid hormone affected the diameter of the seminiferous tubules or II SPC (Figs. 5B, F). Representative photomicrographs of testicular histology (Fig. 6) showed morphological changes parallel to the quantitative measurements in Figs. 4 and 5. In animals implanted for 10 days, no visible differences in germ cell types were seen among treatment groups; I SPG, II SPG, and I SPC were present equally in the testes of all groups (Figs. 6A, B, C). In contrast, 30-day implant with DHT or E_2 _resulted in highly pronounced and visible changes in the histology of the testes (Figs. 6D, E, F). Whereas Ch-treated tubules contained germ cells of all types (Fig. 6D), DHT- and E_2_-treated tubules showed a conspicuous absence of II SPG and I SPC (Figs. 6E, F). The loss of I SPG with DHT and E_2 _treatments was less visible than the loss of other two germ cell types (Figs. 6E, F), a result consistent with the quantitative data (Fig. 5). Interestingly, the formation of spermatozoa appeared to be stimulated by DHT and E_2_; in fact, the most prominent germ cells in tubules of DHT and E_2_-treated were the large bundles of mature spermatozoa, which occupied most of the tubular lumen (Figs. 6E, F).

**Figure 7:**
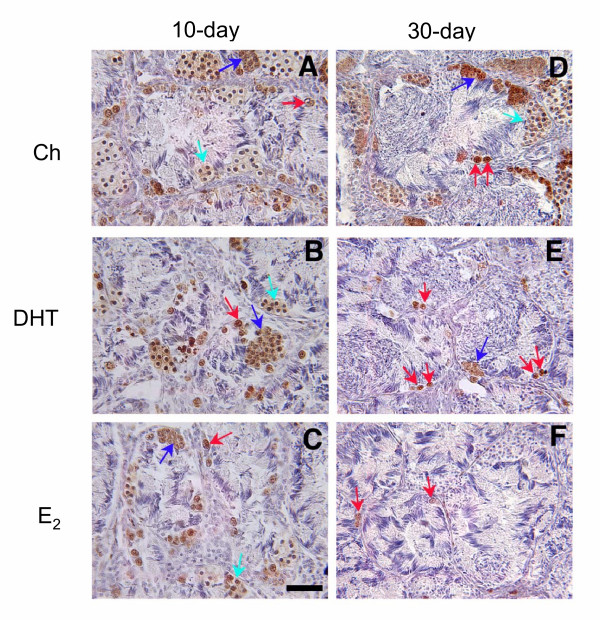
**PCNA ICC on testes of implanted frogs. **Representative photomicrographs of PCNA ICC performed on testicular sections of frogs implanted with **(A, D) **Ch, **(B, E) **DHT, or **(C, F) **E_2_. **(A, B, C) **Testes from animals implanted for 10 days. **(D, E, F) **Testes from animals implanted for 30 days. Brown stain = PCNA immunoreactivity. Blue stain = hematoxylin counterstain. Red arrow = I SPG; dark blue arrow = II SPG; light blue arrow = I SPC. Note DHT and E_2 _had no visible effects on testes of animals implanted for 10 days **(A, B, C)**. In contrast, DHT and E_2 _visibly reduced the presence of PCNA-positive germ cells in animals implanted for 30 days **(D, E, F)**. Σχαλεβαρ = 100 μm.

PCNA ICC was conducted to determine the effects of gonadal steroids on the number of proliferating germ cells and to allow for the more consistent identification of I SPG. In animals implanted with Ch, intense PCNA immuoreactivity was observed in both I SPG and II SPG. In addition, the cytoplasm of I SPC was lightly stained (Figs. 7A, D), since PCNA was also shown to be expressed during the pre-meiotic S phase and meiotic prophase [18]. All spermatogonia (I SPG and II SPG) identifiable by the hematoxylin counterstain were positive for PCNA. Treatment with DHT (Fig. 7B) or E_2 _(Fig. 7C) did not visibly alter the appearance of cells positive for PCNA in frogs implanted for 10 days compared to the Ch control (Fig. 7A). In contrast, in animals implanted for 30 days, a visible reduction in the number of PCNA-positive germ cells was observed in DHT- and E_2_-treated animals (Figs. 7E, F) compared to the Ch control (Fig. 7D). This reduction was primarily due to the decreased presence of I SPG, II SPG, and I SPC in the testes treated with steroid hormones. In control sections incubated with preadsorbed primary antiserum or no primary antiserum, only background staining was present (data not shown).

## Discussion

Under our experimental paradigm, both E_2 _and DHT exerted negative feedback effects upon the reproductive axis of the sexually mature male leopard frogs. The manifestation of these inhibitory effects was time-dependent and required treatment duration for longer than 10 days. In frogs implanted for 30 days, E_2 _decreased circulating LH to undetectable levels and significantly reduced the presence of I SPG, II SPG, and I SPC. The effects of DHT were less potent, reducing only circulating LH, II SPG, and I SPC. Unexpectedly, the presence of post-meiotic germ cells was either unaffected or stimulated with the DHT or E_2 _treatment. These results suggest that along the reproductive axis, the most significant negative feedback effects were upon the pituitary and testes, although a stimulatory effect upon the testes might also exist.

All hormone implants elevated circulating steroid hormones to levels above the Ch controls without exceeding the physiological range reported for male ranid frogs. For instance, depending on the reproductive status, the range of circulating E_2 _reported for sexually mature male ranid frogs was approximately 100 to 3500 pg/ml, and the range of circulating DHT was approximately 1 to 30 ng/ml [6,19]. Another study [20] reported circulating levels of approximately 10 ng/ml for DHT and 2 ng/ml for E_2 _in captive male *R. pipiens*. Thus, circulating E_2 _and DHT in the hormone-implanted animals were largely within the high end of the physiological range.

DHT and E_2 _did not significantly alter hypothalamic GnRH concentration in animals treated for 10 or 30 days. These results were consistent with our previous finding on frogs implanted for 20 days with DHT and E_2_, and speak to the highly stable nature of GnRH peptide accumulation. One should note that this study focused on the mammalian form (Type I) of GnRH because this is the predominant hypophysiotropic form of GnRH in the diencephalon of ranid frogs [21] and the form more sensitive to changes in reproductive status [22]. However, the chicken II (Type II) form of GnRH may also be hypophysiotropic since it binds to pituitary GnRH receptor with high affinity [23], and its presence is detected in the hypothalamic-pituitary portal blood [21]. The ability of steroid hormones to feed back upon the Type II GnRH system in *R. pipiens *is at present unclear and awaits further investigation.

Only E_2 _treatment for the longer duration (30 days) significantly reduced pituitary LH concentration, although a substantial amount of LH still remained in the pituitary glands of these E_2_-treated animals. Thus, the potent suppression of circulating LH in animals treated with E_2 _for 30 days was most likely attributable to the low secretory activity of the pituitary gonadotropes rather than the depletion of LH stores. Somewhat surprising was the inability of E_2 _treatment for 10 days to suppress circulating LH. The pituitaries of ranid frogs were shown to be extremely sensitive to the inhibitory actions of E_2_. In *R. pipiens*, *in vitro *exposure of the pituitary to E_2 _concentrations as low as 100 pg/ml for only 48 hours significantly suppressed both basal and GnRH-stimulated gonadotropin secretion [2]. Extrapolating from this time course and from our current observation that E_2 _implants could elevate circulating E_2 _to about 2000 pg/ml, one might expect a substantial decline in circulating LH of these animals after only 10 days of implant, but this was not the case. It is possible that additional *in vivo *mechanisms exist in these frogs to buffer against short-term estrogenic inhibition. Some of these might include changes in the clearance rate of circulating LH [24] and the levels of sex steroid binding proteins [25]. The former could prolong the half-life of LH in circulation; the latter could dampen the inhibitory effects of E_2 _by binding to E_2 _and decreasing the availability of the bioactive hormone.

Another unexpected observation was that DHT treatment for 30 days also suppressed circulating LH. Our results differ from a previous study demonstrating the inability of DHT to suppress post-gonadectomy rise in LH [2] and showed, for the first time, that DHT participates in the negative feedback regulation of gonadotropin secretion in *R. pipiens*. It is at present unclear if reduced circulating LH is a direct consequence of suppressed secretory activity of the gonadotropes or reduced output from the GnRH system. Based on the previous report that DHT had no direct inhibitory effect on pituitary gonadotropin secretion [2], it seems likely that DHT may achieve negative feedback primarily by targeting the GnRH system to suppress GnRH release. This possibility does not conflict with our current observation that DHT lacks an effect on GnRH content. Since GnRH content is a measure of GnRH peptide accumulation which is a result of transcription, translation, mRNA stability, peptide turnover, or release, it is a poor indicator of GnRH release alone.

One of the most pronounced negative feedback effects of DHT and E_2 _was observed at the level of the testes. In animals implanted for 30 days with these two steroids, a marked loss of several germ cell types (I SPG, II SPG, and I SPC) was observed. Interestingly, although E_2 _exerted a greater disruptive effect on spermatogenesis, similar trends of reduction with virtually no qualitative difference were also observed in DHT-treated testes, suggesting similar outcome might be attained with longer DHT exposure. That DHT- and E_2_-induced disruption of spermatogenesis differed only in the degree of severity suggests the disruption occurred primarily via a common pathway, possibly through the inhibition of gonadotropins. In this study, we could not measure circulating FSH because we lack a homologous FSH RIA. However, previous studies have reported that FSH secretion in *R. pipiens *was under the negative control of E_2 _[2,3] and possibly DHT [2], since gonadectomy significantly elevated the levels of circulating FSH. The observation that spermatogenic disruption occurred only when circulating gonadotropin was reduced (30 day-implants) further lends support to this hypothesis.

Although the roles of FSH and LH in anuran spermatogenesis are not entirely clear, data from other amphibians suggest FSH is essential for supporting the proliferation and survival of spermatogonia [26,27]. Importantly, FSH is required for the completion of the last spermatogonial mitosis, thus the entrance into meiosis and the generation of spermatocytes [27]. On the other hand, LH is specifically required for the stimulation of androgen production in ranid frogs [28] and could be responsible for maintaining high levels of intratesticular androgen required for androgen-dependent stimulation of germ cell formation [29]. Thus, low circulating levels of gonadotropins could be the common pathway leading to defective spermatogenesis in both DHT- and E_2_-treated animals. Although the significant reduction of I SPG in E_2_-treated testes could partially account for the loss of germ cell types that arose from I SPG, this cannot be the sole cause. For example, substantial PCNA-positive I SPG still remained in the testes of frogs implanted with E_2 _for 30 days, yet virtually no II SPG remained in the testes of these animals. Similarly, in 30-day-DHT-treated testes, there was no significant decline in I SPG, but II SPG were markedly reduced. These observations indicate a disproportionate loss of II SPG that may have resulted from their failure to survive. These data were consistent with a previous report in the newt that the mitotic penultimate SPG failed to survive when circulating FSH was suppressed [26]. Taken together, we believe that II SPG was the germ cell type most severely affected by steroid treatments, and the loss of II SPG was the most important underlying cause for disrupted spermatogenesis.

An interesting observation is that although pre-meiotic germ cells (I SPG, II SPG, and I SPC) were adversely affected by steroid hormone implants at 30 days, meiotic or post-meiotic germ cells (II SPC, spermatids and spermatozoa) appeared unaffected or stimulated. In fact, the most conspicuous germ cells in the seminiferous tubules of 30-day E_2_- or DHT-implanted frogs were mature spermatozoa, which occupied the largest bulk of the tubular lumen. It is possible that low circulating FSH had little influence on the germ cells once they entered meiotic division. Under low circulating LH, spermiation was inhibited, and mature spermatozoa continued to accumulate in the tubule. Another possibility is that E_2 _and DHT, while suppressing the presence of pre-meiotic germ cells, actually stimulated the entrance of existing I SPC into meiosis and promoted the survival of post-meiotic germ cells. This possibility was partially supported by the previous observation that testes of frogs treated with DHT for 20 days had fewer II SPG, but significantly more I SPC in the midst of meiotic division [11]. Along the same line of reasoning, it is also possible that DHT and E_2 _facilitated the progression of spermatogenesis to the extent that the intermediate germ cell types could no longer be adequately replenished, leaving tubules filled with post-meiotic cells (primarily spermatozoa) and very little else. Regardless, the trend towards reduced GSI in steroid hormone-treated animals, along with the reduced presence of pre-meiotic germ cells, indicate an overall negative effect of these hormones upon the testes. The abundance of spermatozoa in DHT- and E_2_-treated animals nevertheless raised an interesting possibility for the existence of a positive steroidal effect on spermatogenesis.

Worth mentioning is the possibility that DHT and E_2_, in addition to affecting spermatogenesis by lowering circulating gonadotropins, have also been shown to act directly upon the amphibian testes. Both androgen and estrogen binding sites were found in the amphibian testes [30-33]. A number of physiological responses were presumably mediated through these testicular steroid hormone receptors. For instance, E_2 _acted directly upon the amphibian testes to suppress androgen secretion [34-36], stimulate nuclear translocation of c-Fos [37,38], and enhance proliferation of I SPG [6,37]. Similarly, DHT has also been found to directly modulate androgen secretion [34]. Of interest to the present study is the demonstration that E_2 _directly stimulated SPG I proliferation in *R. esculenta *[6,7,37]. Under our experimental paradigm, however, such a stimulatory effect was not seen in *R. pipiens*. Whether or not this discrepancy was due to the differences in experimental paradigms or species used is at present unclear.

We previously showed that spermatogenesis in the ranid frogs was altered in mature male frogs implanted with DHT and E_2 _for 20 days [11]. Specifically, E_2 _reduced the presence of II SPG and I SPC, whereas DHT reduced only the presence of the former. However, the study represented only a snapshot in time, so no information was available regarding the progression of events that led to the altered formation of germ cells. Moreover, it was unclear if the treatment with these two steroid hormones for shorter or longer periods could impact the testes differently. Our current results showed that both steroids inhibited circulating gonadotropin and disrupted spermatogenesis progressively in a time-dependent manner, with the longer duration of treatment producing the more pronounced effects. Further, the changes in the testes were qualitatively similar between DHT and E_2 _treatments, suggesting declining gonadotropin levels might be the common underlying cause for the disrupted spermatogenesis. These results reflect the highly sensitive nature of the anuran reproductive axis to estrogenic and androgenic modulation. We showed that the continuous exposure of mature frogs to high physiological levels of steroid hormones for a relatively short period could profoundly alter their pituitary and testicular function. In particular, the potency of estrogen hormones raises concerns regarding the potential reproductive disruption that can occur when mature frogs are exposed to short-term and low-level environmental estrogen mimics.

## Authors' contributions

PST designed the experiments, analyzed the data, and prepared the manuscript. AEK and JTJ prepared the silastic capsules, implanted the animals, removed the tissues, and performed all the hormone measurements. KBW implanted some animals, prepared all histological samples, counted the germ cells, and performed the PCNA ICC. All authors read and approved the final manuscript.
